# Association between Braden Score and all-cause mortality in critically ill patients with ischemic stroke: A MIMIC-IV retrospective analysis

**DOI:** 10.1097/MD.0000000000044674

**Published:** 2025-09-26

**Authors:** Yeliang Mei, Jiaqi Zi, Zhenqiang Liu, Jinyu Huang, Zhenyu Fan

**Affiliations:** a Department of Dermatology, Joint Logistics Support Unit No. 904 Hospital, Wuxi, Jiangsu Province, China; b Department of Operation, Joint Logistics Support Unit No. 904 Hospital, Wuxi, Jiangsu Province, China; c Department of Emergency, Joint Logistics Support Unit No. 904 Hospital, Wuxi, Jiangsu Province, China.

**Keywords:** Braden Score, critical care, ischemic stroke, mortality

## Abstract

Ischemic stroke (IS) is the most common subtype of stroke and a leading cause of global morbidity and mortality. The Braden Score (BS), primarily used to assess pressure injury risk, has also been proposed as a marker of patient frailty. This study aimed to evaluate the association between BS and short-term mortality in critically ill patients with IS. Data were extracted from the Medical Information Mart for Intensive Care IV database, identifying IS patients admitted to the intensive care unit (ICU). Patients were categorized into low BS (<16) and high BS (≥16) groups. Propensity score matching was performed to minimize baseline differences. The primary outcomes were 28-day ICU mortality and 90-day in-hospital mortality. Cox proportional hazards models were used to assess associations between BS and mortality, with results reported as hazard ratios (HRs) and 95% confidence intervals (CIs). A total of 2178 patients were included, with 1241 in the low BS group and 937 in the high BS group. Overall, 28-day ICU mortality was 9.23% and 90-day in-hospital mortality was 15.11%. Before and after propensity score matching, low BS was independently associated with higher mortality. After adjusting for confounders, low BS remained significantly associated with increased 28-day ICU mortality (HR: 2.25, 95% CI: 1.34–3.78, *P* = .002) and 90-day in-hospital mortality (HR: 2.10, 95% CI: 1.45–3.05, *P* < .001). BS is significantly associated with short-term mortality in critically ill IS patients and may serve as a simple, noninvasive prognostic tool in ICU settings.

## 1. Introduction

Ischemic stroke (IS) is a major global cause of disability and death, significantly burdening healthcare systems and societies. IS accounts for approximately 70% to 80% of all stroke cases, and its incidence increases markedly with age, especially after the age of 55 years.^[1]^ The most prominent risk factors contributing to IS include diabetes mellitus, hyperlipidemia, smoking, obesity, atrial fibrillation, and sedentary lifestyle.^[2,3]^ Despite advancements in acute stroke management and preventive strategies, the global burden of IS continues to rise due to aging populations and the increasing prevalence of modifiable risk factors.^[4,5]^

The Braden Score (BS), introduced by Bergstrom et al in 1987, has become a widely used and reliable bedside assessment tool in intensive care unit (ICU) for predicting the risk of pressure injuries among critically ill patients.^[6]^ The scale assesses patient conditions in 6 areas: sensory perception, moisture, activity, mobility, nutrition, and friction/shear, offering healthcare providers a practical method independent of laboratory parameters. Beyond its original application for pressure injury prevention, recent studies have demonstrated the broader utility of the BS in predicting various clinical outcomes. Specifically, emerging evidence indicates its predictive value regarding mortality in cardiac intensive care patients,^[7]^ prognosis in hospitalized heart failure and dementia populations,^[8,9]^ delirium risk evaluation,^[10]^ frailty assessment,^[8]^ rehabilitation placement after major surgeries such as pancreatectomy,^[11]^ and the incidence of acute kidney injury following acute coronary syndromes.^[12]^ Furthermore, Ding et al identified the BS as an effective tool for predicting post-stroke pneumonia among IS patients.^[13]^ However, despite these advances, there remains a significant gap regarding the applicability of the BS in predicting ICU and in-hospital all-cause mortality specifically among IS patients.

Due to the significant morbidity and mortality linked to IS, particularly among critically ill patients, developing a dependable prognostic marker could enable early detection of high-risk individuals, ensure timely interventions, and enhance clinical management. Therefore, this study aims to explore the relationship between the BS and both ICU and in-hospital all-cause mortality in patients admitted with IS, potentially expanding the clinical utility of this simple yet informative bedside assessment tool.

## 2. Materials and methods

### 2.1. Study participants

This retrospective study analyzed clinical data from the Medical Information Mart for Intensive Care IV (MIMIC-IV) (version 2.0) database, a comprehensive resource maintained by the Massachusetts Institute of Technology Computational Physiology Laboratory. MIMIC-IV comprises comprehensive, high-quality medical records of patients admitted to the ICUs at Beth Israel Deaconess Medical Center.^[14]^ Data collection and curation were approved by the Institutional Review Board of Beth Israel Deaconess Medical Center, which waived the need for individual informed consent in support of the data-sharing initiative. As a result, no further ethical approval was required for this study. Yeliang Mei registered, completed training, and received authorization to access the MIMIC-IV database. Informed consent was unnecessary for data collection since all patient data in the database is anonymized to maintain privacy and confidentiality.

**Figure 1.**
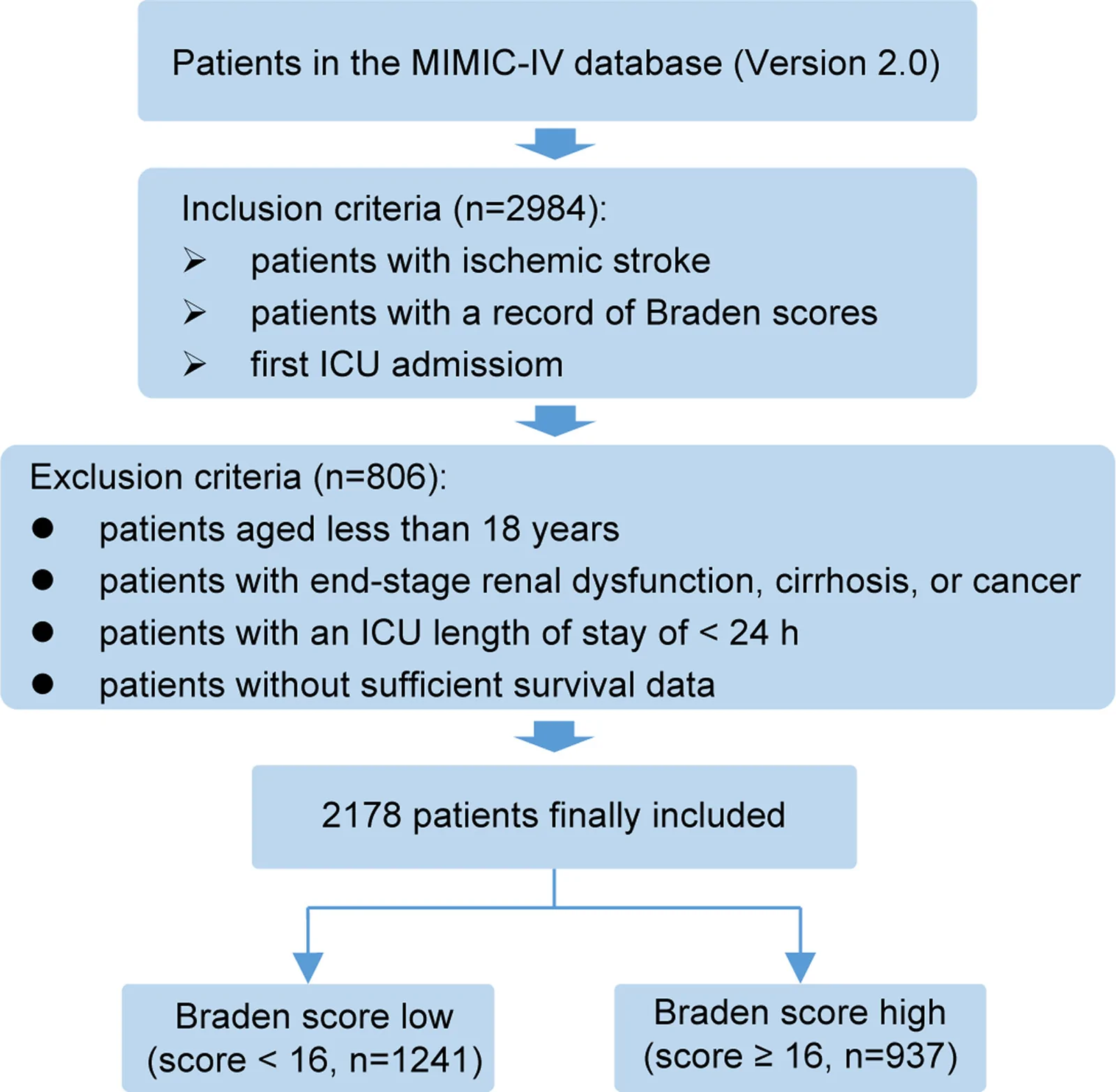
Flow chart of patient selection in this study.

Patients with IS were identified in the MIMIC-IV database using International Classification of Diseases (ICD)-9 and ICD-10 diagnostic codes. Patients with IS were identified using ICD-9 codes 433, 434, 436, 437.0, 437.1, or ICD-10 codes I63, I65, and I66.^[15]^ The exclusion criteria included: first, patients under 18 years at initial admission; second, those with end-stage renal dysfunction, liver cirrhosis, or cancer; third, ICU stays shorter than 24 hours; fourth, incomplete survival data. Ultimately, 2178 eligible patients were included and categorized into the low BS group (BS < 16, n = 1241) and the high BS group (BS ≥ 16, n = 937) (Fig. 1).

### 2.2. Data collection

Data extraction utilized PostgreSQL 13.7.2 and Navicat Premium 16 with structured query language. The extracted variables wee grouped into 4 categories: first, patient demographics, encompassing age and gender; second, clinical scores, including the Sequential Organ Failure Assessment (SOFA),^[16]^ Acute Physiology Score III (APS-III), Simplified Acute Physiology Score II (SAPS-II), Oxford Acute Severity of Illness Score (OASIS), Glasgow Coma Scale (GCS), and Charlson Comorbidity Index (CCI); third, comorbidities, such as heart failure, myocardial infarction, atrial fibrillation, respiratory failure, diabetes, paraplegia, renal disease, and sepsis; fourth, laboratory measurements, comprising white blood cell count (WBC), red blood cell count (RBC), platelet count, hemoglobin level, sodium, serum creatinine, and glucose. The follow-up period spanned from ICU admission to the date of death. The BS at ICU admission served as the exposure variable, representing the initial recorded score upon ICU entry.^[17]^ This scale assesses pressure injury risk across 6 domains: sensory perception, moisture, activity, mobility, nutrition, and friction/shear (Table S1, Supplemental Digital Content, https://links.lww.com/MD/Q59). Scores range from 6 to 23, where lower scores signify an increased risk of pressure injuries.^[18]^ The primary endpoints of this study were 28-day ICU mortality and 90-day in-hospital mortality.

Variables containing more than 20% missing values were excluded from analysis. Missing data for variables with fewer than 20% missing observations were imputed using multiple imputation via random forest modeling, performed with the “mice” package in R software (R Foundation for Statistical Computing, Vienna, Austria)^[19]^ (Table S2, Supplemental Digital Content, https://links.lww.com/MD/Q59).

### 2.3. Propensity score matching (PSM) analysis

To reduce potential selection bias and confounding factors between groups, we performed PSM.^[20]^ Propensity scores were calculated via logistic regression, using group assignment as the dependent variable and baseline characteristics (including age, gender, SOFA, APS-III, SAPS-II, OASIS, GC, CCI, and clinical conditions like heart failure, myocardial infarction, atrial fibrillation, respiratory failure, diabetes, paraplegia, renal disease, and sepsis) as independent variables. The propensity model incorporated laboratory parameters such as WBC and RBC counts, platelet count, hemoglobin concentration, serum sodium, serum creatinine, and glucose levels. A nearest-neighbor algorithm with a 1:1 ratio and a caliper width of 0.2 standard deviations of the logit of the propensity score was used to achieve optimal matching quality and minimize imbalance. Post-matching, group balance was evaluated using standardized mean differences for baseline covariates, with a standardized mean difference below 0.1 signifying sufficient balance.

### 2.4. Statistical analysis

The Shapiro–Wilk test assessed the normality of continuous variables, revealing that most did not follow a normal distribution. Continuous variables were summarized as medians with interquartile ranges, and group comparisons were performed using the Wilcoxon rank-sum test. Categorical variables were expressed as frequencies and percentages, and intergroup differences were evaluated using the Chi-square test. Kaplan–Meier curves depicted the 28-day ICU and 90-day in-hospital survival probabilities between groups, with the log-rank test assessing survival differences. Cox proportional hazards regression models assessed the relationship between the BS and mortality, presenting results as hazard ratios (HR) with 95% confidence intervals (CI). Subgroup analyses were conducted to examine the relationship between BS and mortality across subpopulations categorized by age, gender, heart failure, myocardial infarction, atrial fibrillation, respiratory failure, diabetes, paraplegia, and renal disease. A 2-sided *P*-value below .05 was deemed statistically significant. R (version 4.2.1) was used for statistical analyses.

## 3. Results

### 3.1. Patient characteristics

In total, 2178 critically ill patients diagnosed with IS were enrolled in this study. The median age was 70.69 years (interquartile range [IQR]: 59.29–81.48 years), and 409 (50.28%) patients were male (Table 1). After PSM, 1282 critically ill IS patients remained for analysis, equally divided into low BS (n = 641) and high BS (n = 641) groups. Among these matched patients, the median age was 70.83 years (IQR: 60.10–81.10 years), with 409 (50.08%) being male (Table S3, Supplemental Digital Content, https://links.lww.com/MD/Q59).

**Table 1:** Characteristics and outcomes of participants categorized by Braden Scores before propensity score matching.

Variable	Total (n = 2178)	Braden Score low (n = 1241)	Braden Score high (n = 937)	*P*-value
Personal characteristics				
Age (yr)	70.67 (59.29, 81.48)	72.95 (61.76, 83.17)	67.78 (56.41, 79.51)	<.001
Gender (%)				.009
Female	1083 (49.72)	654 (52.70)	429 (45.78)	
Male	1095 (50.28)	587 (47.30)	508 (54.22)	
Scores				
SOFA	3.00 (2.00, 6.00)	4.00 (3.00, 7.00)	2.00 (1.00, 4.00)	<.001
APS-III	38.00 (28.00, 51.00)	44.00 (33.00, 58.00)	32.00 (24.00, 41.00)	<.001
SAPS-II	33.00 (26.00, 42.00)	37.00 (31.00, 47.00)	28.00 (20.00, 35.00)	<.001
OASIS	32.00 (26.00, 38.00)	35.00 (29.00, 40.00)	28.00 (23.00, 33.00)	<.001
GCS	14.00 (12.00, 15.00)	14.00 (10.00, 15.00)	14.00 (14.00, 15.00)	<.001
CCI	6.00 (4.00, 8.00)	7.00 (5.00, 8.00)	6.00 (4.00, 7.00)	<.001
Comorbidities				
Heart failure (%)				<.001
No	1623 (74.52)	873 (70.35)	750 (80.04)	
Yes	555 (25.48)	368 (29.65)	187 (19.96)	
Myocardial infarction (%)				<.01
No	1814 (83.29)	1007 (81.14)	807 (86.13)	
Yes	364 (16.71)	234 (18.86)	130 (13.87)	
Atrial fibrillation (%)				<.001
No	1309 (60.10)	680 (54.79)	629 (67.13)	
Yes	869 (39.90)	561 (45.21)	308 (32.87)	
Respiratory failure (%)				.021
No	1815 (83.33)	1014 (81.71)	801 (85.49)	
Yes	363 (16.67)	227 (18.29)	136 (14.51)	
Diabetes (%)				.008
No	1483 (68.09)	813 (65.51)	670 (71.50)	
Yes	695 (31.91)	428 (34.49)	267 (28.50)	
Paraplegia (%)				<.001
No	1042 (47.84)	548 (44.16)	494 (52.72)	
Yes	1136 (52.16)	693 (55.84)	443 (47.28)	
Renal disease (%)				<.001
No	1787 (82.05)	988 (79.61)	799 (85.27)	
Yes	391 (17.95)	253 (20.39)	138 (14.73)	
Sepsis (%)				<.001
No	1891 (86.82)	1007 (81.14)	884 (94.34)	
Yes	287 (13.18)	234 (18.86)	53 (5.66)	
Laboratory tests				
WBC (10^9^/L)	10.30 (8.00, 13.90)	11.40 (8.50, 15.00)	9.20 (7.20, 12.00)	<.001
RBC (10^9^/L)	3.94 (3.38, 4.40)	3.79 (3.25, 4.31)	4.05 (3.58, 4.53)	<.001
Platelet (10^9^/L)	205.00 (156.00, 260.00)	198.00 (149.00, 256.00)	212.00 (170.00, 266.00)	<.001
Haemoglobin (g/dL)	11.70 (10.00, 13.20)	11.20 (9.70, 12.90)	12.20 (10.60, 13.60)	<.001
Sodium (mEq/L)	140.00 (137.00, 142.00)	140.00 (138.00, 143.00)	139.00 (137.00, 141.00)	<.001
Serum creatinine (mg/dL)	0.90 (0.70, 1.30)	1.00 (0.80, 1.50)	0.90 (0.70, 1.10)	<.001
Glucose (mg/dL)	124.00 (103.00, 157.00)	133.00 (110.00, 171.00)	111.00 (98.00, 141.00)	<.001
Outcomes				
ICU LOS (d)	3.65 (1.86, 7.57)	4.84 (2.33, 9.31)	2.60 (1.37, 5.16)	<.001
Hospital LOS (d)	8.94 (4.86, 18.09)	11.97 (6.24, 21.93)	6.78 (3.73, 12.90)	<.001
28‐day ICU mortality (%)				<.001
Alive	1977 (90.77)	1063 (85.66)	914 (97.55)	
Death	201 (9.23)	178 (14.34)	23 (2.45)	
90‐day hospital mortality (%)				<.001
Alive	1849 (84.89)	959 (77.28)	890 (94.98)	
Death	329 (15.11)	282 (22.72)	47 (5.02)	

Medians and interquartile ranges (25th and 75th percentiles) were calculated for continuous variables and frequencies and percentages for categorical variables. The Wilcoxon rank-sum test was used to compare group differences for continuous variables and Chi-squared tests for categorical variables.

APS-III = Acute Physiology Score III, CCI = Charlson Comorbidity Index, GCS = Glasgow Coma Scale, ICU = intensive care unit, LOS = length of stay, OASIS = Oxford Acute Severity of Illness Score, RBC = red blood cell, SAPS-II = Simplified Acute Physiological Score II, SOFA = Sequential Organ Failure Assessment, WBC = white blood cell.

The low BS group had a significantly older median age (72.95 vs 67.78 years; *P* < .001) and a higher percentage of females (52.70% vs 45.78%; *P* = .009). Clinical severity scores such as SOFA, APS-III, SAPS-II, OASIS, GCS, and CCI demonstrated significantly higher disease severity in the low BS group compared to the high BS group (all *P* < .001). The low BS group exhibited significantly higher prevalence rates of various comorbidities compared to the control group: heart failure (29.65% vs 19.96%; *P* < .001), myocardial infarction (18.86% vs 13.87%; *P* < .01), atrial fibrillation (45.21% vs 32.87%; *P* < .001), respiratory failure (18.29% vs 14.51%; *P* = .021), diabetes (34.49% vs 28.50%; *P* = .008), paraplegia (55.84% vs 47.28%; *P* < .001), renal disease (20.39% vs 14.73%; *P* < .001), and sepsis (18.86% vs 5.66%; *P* < .001). Laboratory tests indicated that the low BS group had significantly elevated WBC counts, serum creatinine, and glucose levels, alongside reduced RBC counts, platelet counts, and hemoglobin levels (all *P* < .001). Patients with low BS experienced significantly longer ICU stays (median: 4.84 vs 2.60 days; *P* < .001) and hospital stays (median: 11.97 vs 6.78 days; *P* < .001). The low BS group exhibited significantly higher 28-day ICU mortality (14.34% vs 2.45%; *P* < .001) and 90-day in-hospital mortality (22.72% vs 5.02%; *P* < .001) (Table 1).

After PSM, groups showed no significant differences in age (median 71.13 vs 70.49 years; *P* = .392), gender distribution (*P* = .544), or clinical severity scores (SOFA, APS-III, SAPS-II, OASIS, GCS, and CCI; all *P* > .05). The prevalence of comorbidities such as heart failure, myocardial infarction, atrial fibrillation, respiratory failure, diabetes, paraplegia, renal disease, and sepsis was similar across groups (all *P* > .05). No significant differences were observed in laboratory parameters, including WBC count, RBC count, platelet count, hemoglobin, sodium, serum creatinine, and glucose levels (all *P* > .05). However, significant differences remained in clinical outcomes: patients in the low BS group had longer ICU stays (median: 4.09 vs 2.87 days; *P* < .001) and hospital stays (median: 9.91 vs 7.77 days; *P* = .009), with significantly higher mortality rates at both 28-day ICU (9.98% vs 3.43%; *P* < .001) and 90-day in-hospital endpoints (15.44% vs 6.86%; *P* < .001) (Table S3, Supplemental Digital Content, https://links.lww.com/MD/Q59).

### 3.2. BS and subscores

**Figure 2.**
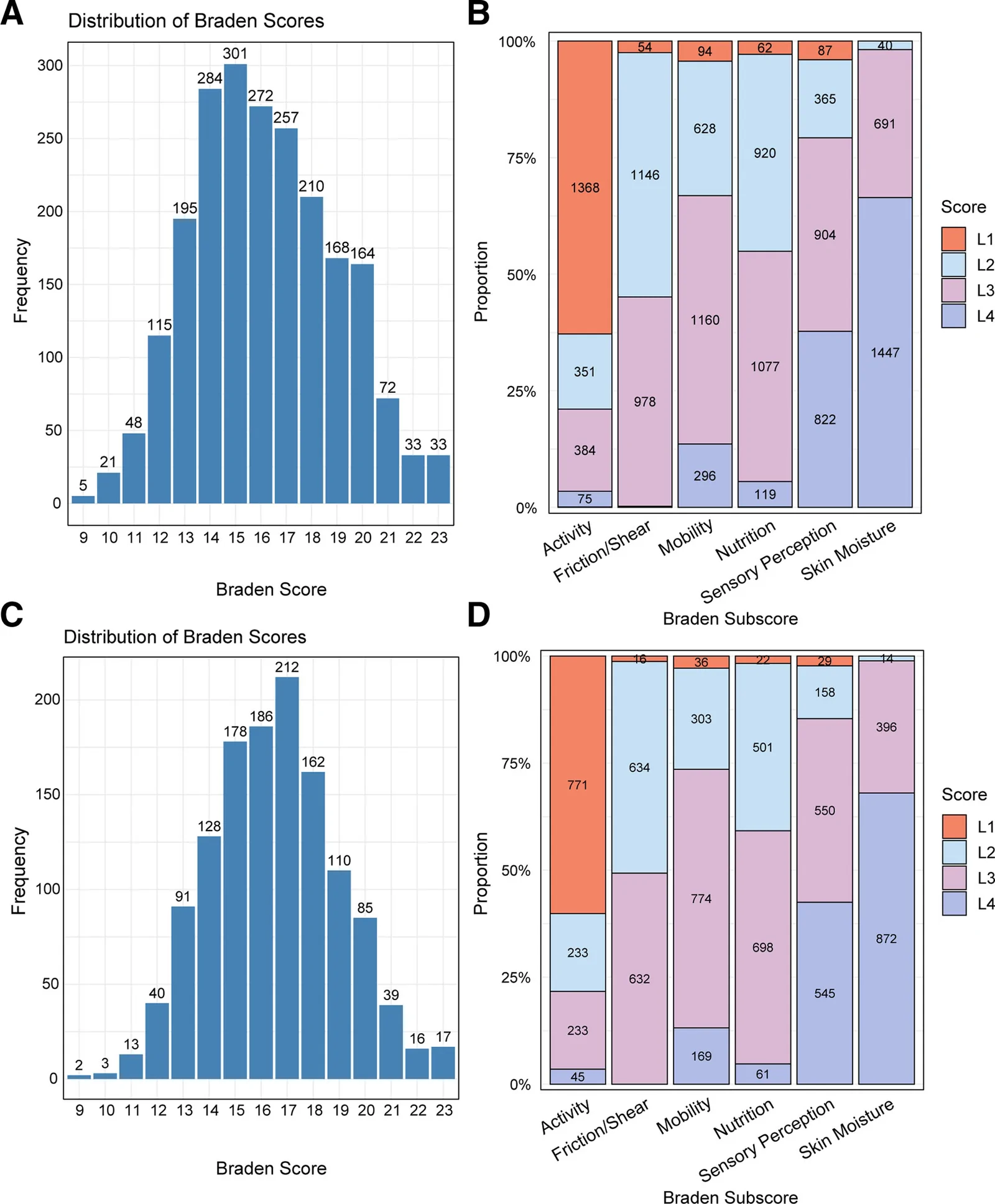
Distribution of individual Braden Scores and subscores across the full cohort. (A) Distribution of individual Braden Score before propensity score matching. (B) Distribution of individual Braden subscore before propensity score matching. (C) Distribution of individual Braden Score after propensity score matching. (D) Distribution of individual Braden subscore after propensity score matching.

Figure 2 illustrates the distribution of BS and their respective subscores both before and after PSM. Before PSM, the median BS among patients was 16 (IQR: 14–18), indicating a relatively low overall score (Fig. 2A). Analyzing the individual subscores revealed that, apart from the moisture subscore, which was generally adequate, the remaining subscores tended to be low. Specifically, over half of the patients were confined to bed with limited activity levels (Fig. 2B). After PSM, patients showed a similar distribution pattern, with a median BS of 16.5 (IQR: 15–18), again reflecting a lower score profile (Fig. 2C). Consistent with the pre-PSM observations, the moisture subscore remained adequate, whereas the other subscores remained low. Notably, more than 50% of these patients were also bedridden with severely restricted activities (Fig. 2D).

### 3.3. Primary outcomes

**Figure 3.**
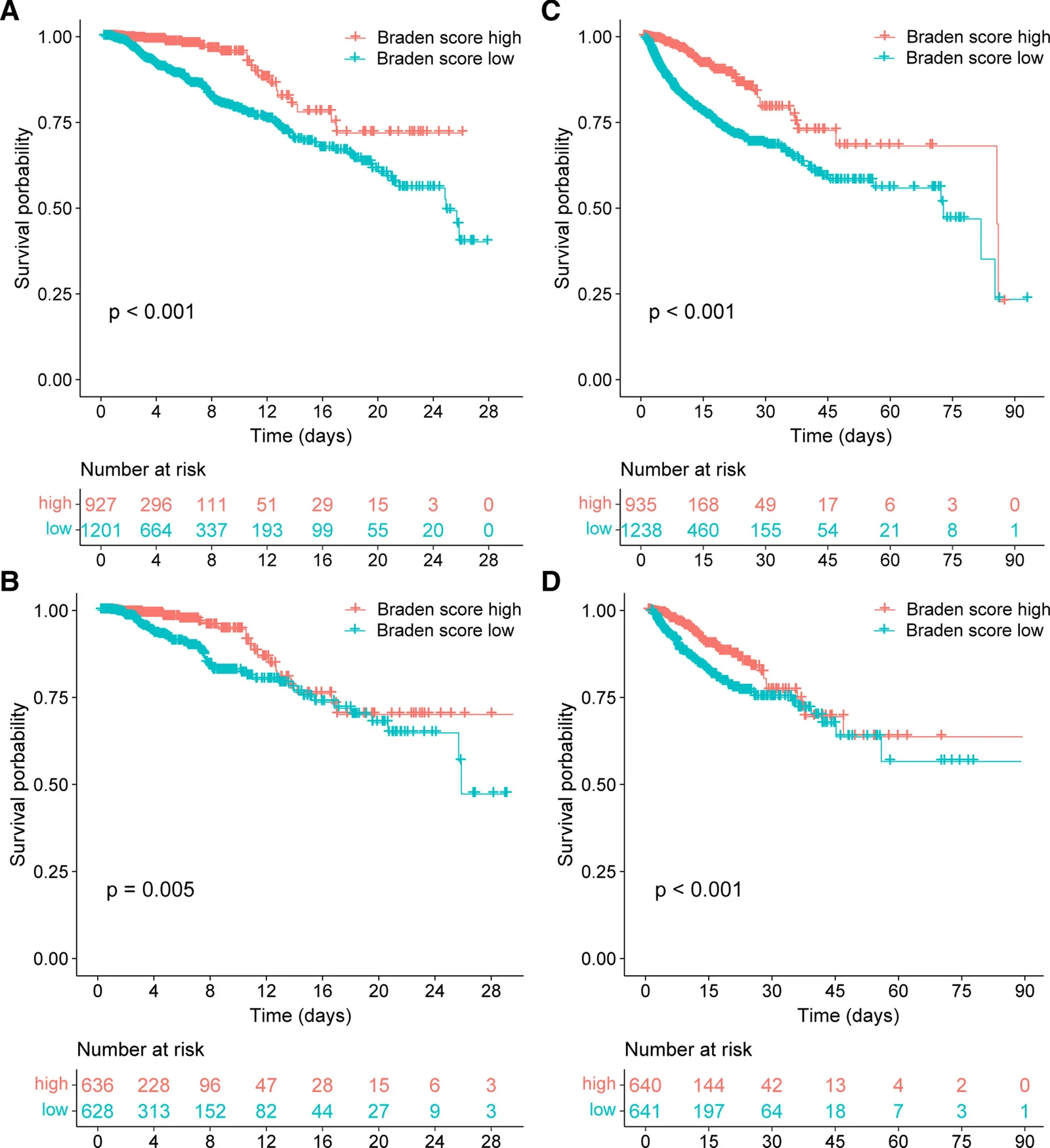
Kaplan–Meier survival analysis curves for all-cause mortality between Braden Score high group and low group. Kaplan–Meier survival analysis curves for 28-day ICU mortality before (A) and after (B) propensity score matching. Kaplan–Meier survival analysis curves for 90-day in-hospital mortality before (C) and after (D) propensity score matching.

Kaplan–Meier survival curves (Fig. 3) demonstrated significantly reduced survival probabilities for patients in the low BS group compared to the high BS group, both pre- and post-PSM. For 28-day ICU mortality, patients with low BS exhibited significantly lower survival rates both prior to (log-rank *P* < .001, Fig. 3A) and following PSM (log-rank *P* = .005, Fig. 3B). The low BS group demonstrated significantly reduced survival probabilities for 90-day hospital mortality both pre-PSM (log-rank *P* < .001, Fig. 3C) and post-PSM (log-rank *P* < .001, Fig. 3D).

Table 2 presents the Cox proportional hazards regression analysis results assessing the link between BS and all-cause mortality prior to PSM. Lower BS consistently correlated with significantly increased risks for both 28-day ICU and 90-day hospital mortality across all models. Each unit increase in continuous BS significantly reduced mortality risks (*P* < .001). In Model 5, a unit increase in BS significantly decreased the risk of 28-day ICU mortality (adjusted HR: 0.87, 95% CI: 0.82–0.94, *P* < .001) and 90-day hospital mortality (adjusted HR: 0.85, 95% CI: 0.80–0.90, *P* < .001). Patients in the low BS group faced significantly higher mortality risks than those in the high BS group for both 28-day ICU mortality (adjusted HR: 2.22, 95% CI: 1.40–3.52, *P* < .001) and 90-day hospital mortality (adjusted HR: 2.34, 95% CI: 1.68–3.26, *P* < .001).

**Table 2:** Cox proportional hazard ratios (HR) for all-cause mortality before propensity score matching.

Categories	Model 1		Model 2		Model 3		Model 4		Model 5	
	HR (95% CI)	*P*-value	HR (95% CI)	*P*-value	HR (95% CI)	*P*-value	HR (95% CI)	*P*-value	HR (95% CI)	*P*-value
28‐day ICU mortality (continuous variable)	0.81 (0.76–0.86)	<.001	0.80 (0.75–0.86)	<.001	0.86 (0.80–0.92)	<.001	0.86 (0.80–0.92)	<.001	0.87 (0.82–0.94)	<.001
90‐day hospital mortality (continuous variable)	0.81 (0.78–0.85)	<.001	0.80 (0.77–0.84)	<.001	0.85 (0.80–0.89)	<.001	0.85 (0.80–0.89)	<.001	0.85 (0.80–0.90)	<.001
28‐day ICU mortality (binary variable)										
Braden Score high	Ref		Ref		Ref		Ref		Ref	
Braden Score low	3.16 (2.05–4.90)	<.001	2.97 (1.92–4.60)	<.001	2.41 (1.54–3.77)	<.001	2.39 (1.52–3.74)	<.001	2.22 (1.40–3.52)	<.001
90‐day hospital mortality (binary variable)										
Braden Score high	Ref		Ref		Ref		Ref		Ref	
Braden Score low	3.05 (2.24–4.16)	<.001	2.88 (2.11–3.93)	<.001	2.30 (1.67–3.18)	<.001	2.31 (1.67–3.20)	<.001	2.34 (1.68–3.26)	<.001

Model 1: unadjusted.

Model 2: adjusted for age, gender.

Model 3: adjusted for age, gender, SOFA, APS-III, SAPS-II, OASIS, GCS, CCI.

Model 4: adjusted for age, gender, SOFA, APS-III, SAPS-II, OASIS, GCS, CCI, heart failure, myocardial infarction, atrial fibrillation, respiratory failure, diabetes, paraplegia, renal disease, sepsis.

Model 5: adjusted for age, gender, SOFA, APS-III, SAPS-II, OASIS, GCS, CCI, heart failure, myocardial infarction, atrial fibrillation, respiratory failure, diabetes, paraplegia, renal disease, sepsis, WBC, RBC, platelet, hemoglobin, sodium, serum creatinine, glucose.

APS-III = Acute Physiology Score III, CCI = Charlson Comorbidity Index, GCS = Glasgow Coma Scale, ICU = intensive care unit, OASIS = Oxford Acute Severity of Illness Score, RBC = red blood cell, Ref = reference, SAPS-II = Simplified Acute Physiological Score II, SOFA = Sequential Organ Failure Assessment, WBC = white blood cell.

Table 3 presents the Cox proportional hazards regression analyses assessing the link between BS and all-cause mortality following PSM. Similarly, lower BS consistently correlated with significantly increased risks of both 28-day ICU and 90-day hospital mortality across all models. Each unit increase in continuous BS significantly reduced mortality risks (*P* < .05). In Model 5, a unit increase in BS was associated with significantly reduced risks of 28-day ICU mortality (adjusted HR: 0.88, 95% CI: 0.80–0.97, *P* = .011) and 90-day hospital mortality (adjusted HR: 0.85, 95% CI: 0.79–0.92, *P* < .05). Patients in the low BS group exhibited significantly higher mortality risks than those in the high BS group for both 28-day ICU mortality (adjusted HR: 2.25, 95% CI: 1.34–3.78, *P* = .002) and 90-day hospital mortality (adjusted HR: 2.10, 95% CI: 1.45–3.05, *P* < .001).

**Table 3:** Cox proportional hazard ratios (HR) for all-cause mortality after propensity score matching.

Categories	Model 1		Model 2		Model 3		Model 4		Model 5	
	HR (95% CI)	*P*-value	HR (95% CI)	*P*-value	HR (95% CI)	*P*-value	HR (95% CI)	*P*-value	HR (95% CI)	*P*-value
28‐day ICU mortality (Continuous variable)	0.87 (0.80–0.95)	.002	0.86 (0.78–0.94)	<.001	0.87 (0.80–0.95)	.003	0.87 (0.79–0.95)	.002	0.88 (0.80–0.97)	.011
90‐day hospital mortality (Continuous variable)	0.85 (0.80–0.91)	<.001	0.84 (0.78–0.90)	<.001	0.86 (0.80–0.92)	<.001	0.85 (0.79–0.91)	<.001	0.85 (0.79–0.92)	<.001
28‐day ICU mortality (Binary variable)										
Braden Score high	Ref		Ref		Ref		Ref		Ref	
Braden Score low	1.96 (1.21–3.19)	.012	2.04 (1.25–3.32)	.004	2.20 (1.34–3.60)	.002	2.22 (1.35–3.67)	.002	2.25 (1.34–3.78)	.002
90‐day hospital mortality (Binary variable)										
Braden Score high	Ref		Ref		Ref		Ref		Ref	
Braden Score low	1.83 (1.28–2.61)	<.001	1.89 (1.32–2.70)	<.001	1.97 (1.37–2.82)	<.001	1.98 (1.38–2.84)	<.001	2.10 (1.45–3.05)	<.001

Model 1: unadjusted.

Model 2: adjusted for age, gender.

Model 3: adjusted for age, gender, SOFA, APS-III, SAPS-II, OASIS, GCS, CCI.

Model 4: adjusted for age, gender, SOFA, APS-III, SAPS-II, OASIS, GCS, CCI, heart failure, myocardial infarction, atrial fibrillation, respiratory failure, diabetes, paraplegia, renal disease, sepsis.

Model 5: adjusted for age, gender, SOFA, APS-III, SAPS-II, OASIS, GCS, CCI, heart failure, myocardial infarction, atrial fibrillation, respiratory failure, diabetes, paraplegia, renal disease, sepsis, WBC, RBC, platelet, hemoglobin, sodium, serum creatinine, glucose.

APS-III = Acute Physiology Score III, CCI = Charlson Comorbidity Index, GCS = Glasgow Coma Scale, ICU = intensive care unit, OASIS = Oxford Acute Severity of Illness Score, RBC = red blood cell, Ref = reference, SAPS-II = Simplified Acute Physiological Score II, SOFA = Sequential Organ Failure Assessment, WBC = white blood cell.

### 3.4. Subgroup analysis

**Figure 4.**
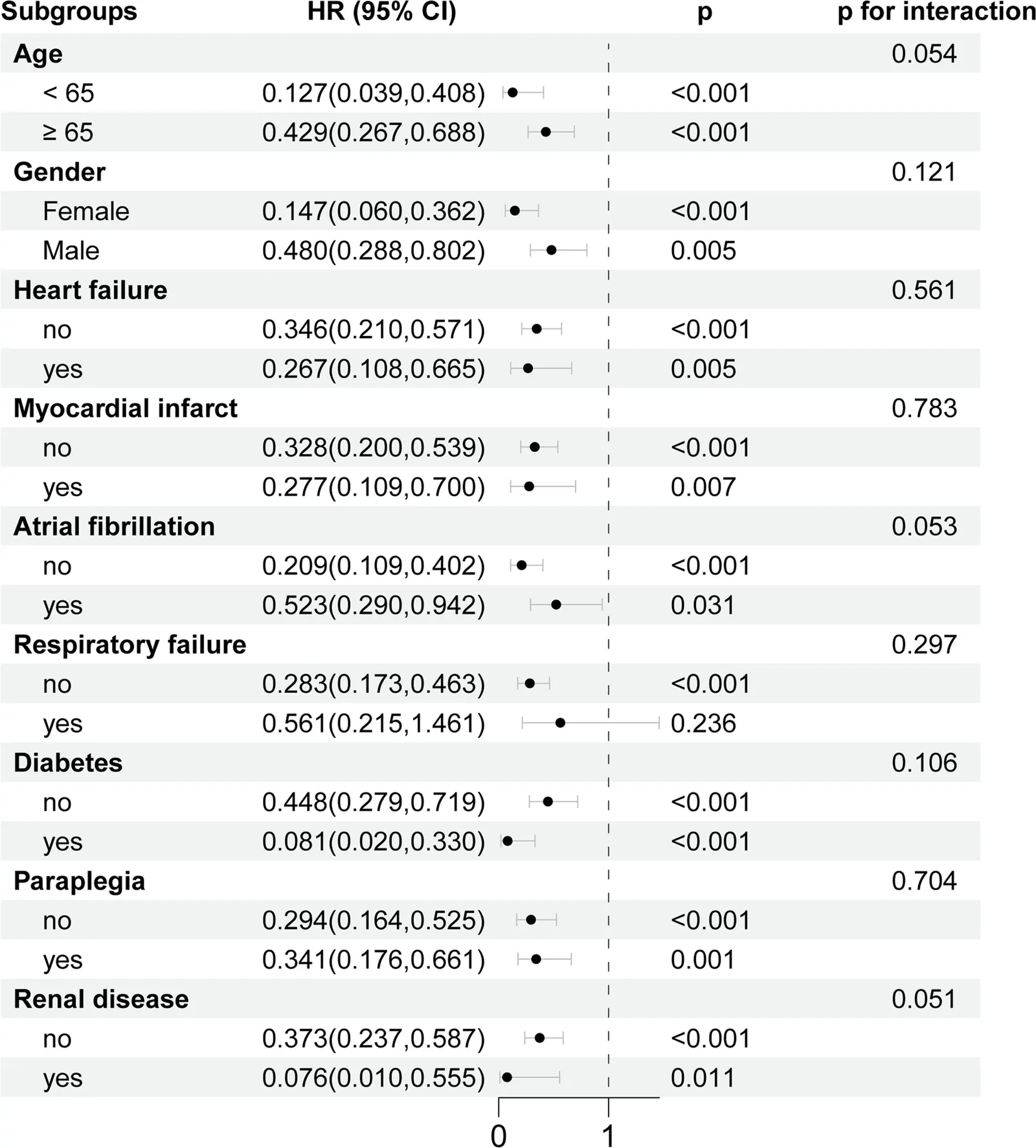
Forest plots of hazard ratios for 28-day ICU mortality in different subgroups. CI = confidence interval, HR = hazard ratio.

Cox regression subgroup analysis for 28-day ICU mortality, using the low BS group as a reference, revealed significantly lower mortality risks for patients with high BS across various clinical subgroups. Specifically, younger patients (<65 years) exhibited a more pronounced predictive value (HR: 0.13, 95% CI: 0.04–0.41) compared to older patients (≥65 years; HR: 0.43, 95% CI 0.27–0.69), with the interaction term approaching statistical significance (*P* = .054). The predictive value was more pronounced in females (HR: 0.15, 95% CI: 0.06–0.36) compared to males (HR: 0.48, 95% CI: 0.29–0.80), although the interaction did not reach statistical significance (*P* = .121). Significant predictive values were consistent across subgroups divided by heart failure, myocardial infarction, atrial fibrillation, respiratory failure, diabetes, paraplegia, and renal disease, with no notable heterogeneity (interaction *P*-values > .05) (Fig. 4).

**Figure 5.**
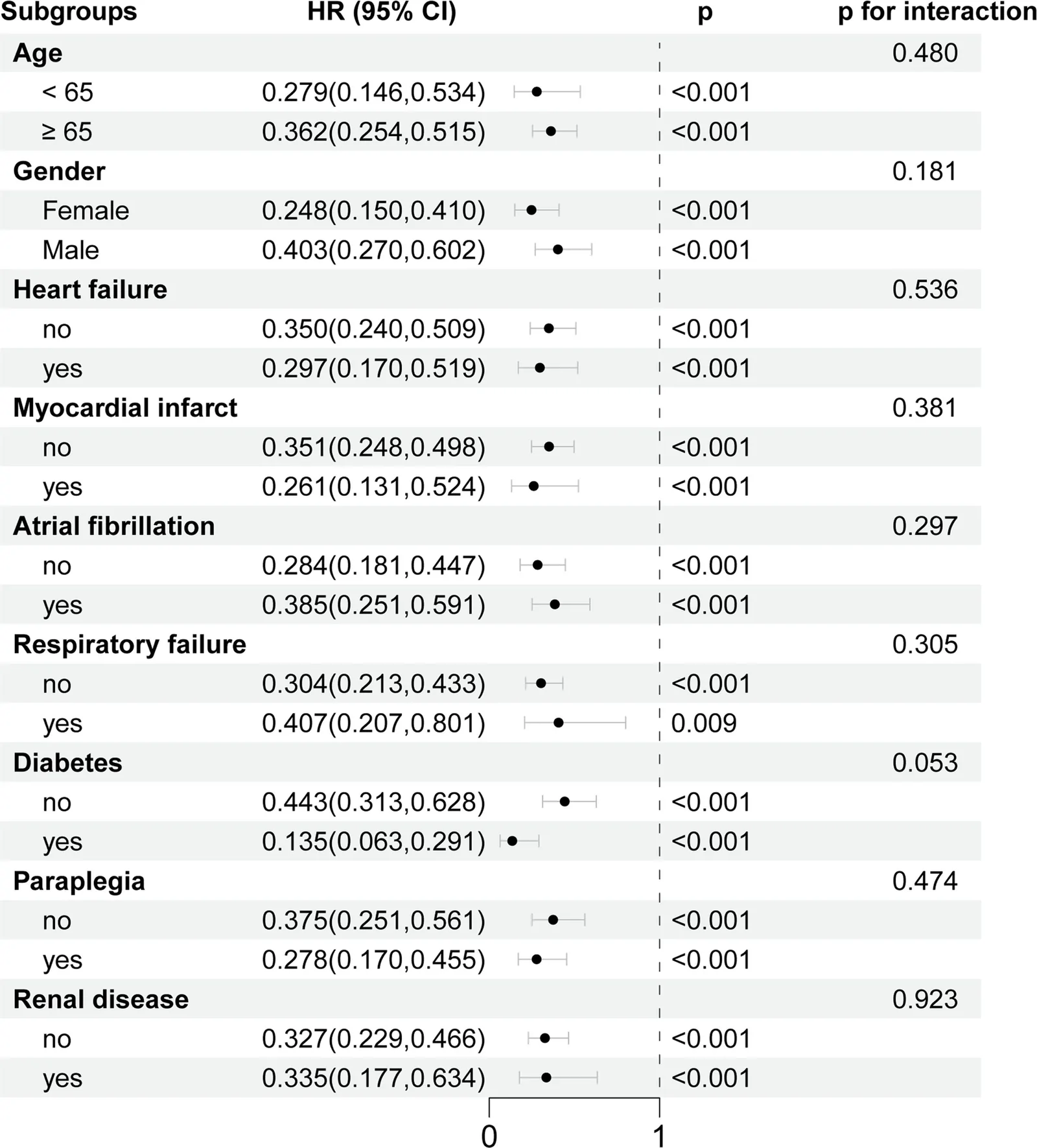
Forest plots of hazard ratios for 90-day in-hospital mortality in different subgroups. CI = confidence interval, HR = hazard ratio.

The subgroup analysis for 90-day hospital mortality (with the low BS group as the reference) similarly demonstrated consistent predictive values for patients in the high BS group across all evaluated clinical subgroups (all *P*-values < .05). Additionally, no significant subgroup heterogeneity was identified in analyses stratified by gender, heart failure, myocardial infarction, atrial fibrillation, respiratory failure, diabetes, paraplegia, or renal disease (all interaction *P*-values > .05) (Fig. 5).

## 4. Discussion

This retrospective study evaluated the association between BS and all-cause mortality in critically ill IS patients using MIMIC-IV database data. Analysis of 2178 eligible patients revealed that a low BS at ICU admission significantly correlated with higher risks of 28-day ICU mortality and 90-day in-hospital mortality. These relationships remained robust after adjustment for confounders and were further confirmed in propensity score-matched cohorts. Notably, subgroup analyses revealed consistent prognostic value of BS across various clinical subgroups, with no substantial effect modification. By applying PSM to minimize selection bias and balance baseline characteristics, this study strengthens the validity of the observed associations and highlights the potential utility of BS as a simple, bedside predictor of mortality risk in critically ill IS patients.

Given the high rates of disability and mortality associated with IS, early identification of high-risk patients is essential for optimizing clinical management and improving outcomes. Accordingly, the development of reliable and accessible prognostic indicators is critical for guiding timely interventions in critical care settings. A comprehensive systematic review by Montellano et al identified 257 biomarkers across 291 studies, several of which were consistently associated with poor outcomes in IS patients.^[21]^ Prominent biomarkers include natriuretic peptides (BNP, NT-proBNP), copeptin, cortisol, procalcitonin, mannose-binding lectin, and adipocyte fatty acid-binding protein. These markers reflect diverse pathophysiological processes, including cardiac dysfunction, neuroendocrine stress, systemic inflammation, and atherogenesis. Notably, copeptin and cortisol demonstrated additive prognostic value beyond conventional clinical models, while natriuretic peptides showed strong and consistent associations with mortality, particularly in cardioembolic stroke. Inflammatory markers such as procalcitonin and mannose-binding lectin also exhibited prognostic potential, although their associations may be confounded by concurrent infections.^[21]^ Adipocyte fatty acid-binding protein was identified as a significant independent predictor of mortality and adverse functional outcomes. Despite these promising results, several methodological limitations persist in the biomarker literature, including inconsistent confounder adjustment, limited external validation, and heterogeneity in measurement and analysis. These issues highlight the need for standardized, well-controlled studies to validate and compare biomarker performance. The National Institutes of Health Stroke Scale is a reliable predictor of in-hospital mortality and poor functional recovery, with higher admission scores indicating worse outcomes.^[22]^ Beyond these established tools, the BS, originally developed to assess pressure injury risk, has recently gained attention as a potential prognostic indicator in critically ill IS patients. Retrospective ICU-based studies have shown that lower BS at admission is independently associated with increased a higher risk of delirium, even after adjusting for age, sex, and comorbidities.^[23]^

Recent studies have increasingly emphasized the expanding clinical utility of the BS beyond its original role. A growing body of evidence supports a strong association between lower BS values and adverse outcomes, including mortality, frailty, delirium, and infection risk. For example, Tang et al found that BS ≤ 15.5 was significantly linked to higher 30-day mortality in ICU patients with IS, maintaining strong predictive accuracy after adjusting for confounders.^[15]^ Similarly, Li et al found that low BS were identified as independent predictors of delirium and heightened 90- and 180-day mortality in sepsis patients, highlighting the score’s significant prognostic value.^[24]^ Cheng et al conducted a study on critically ill patients with dementia and the demonstrated that BS was independently associated with 90-day mortality, as well as higher risks of aspiration pneumonia and pressure injuries.^[9]^ The psychometric properties of BS in ICU settings have been reviewed systematically by Mehicic et al, who confirmed moderate-to-good reliability and convergent validity, though optimal cutoff values varied across studies.^[25]^ Wang et al further proposed BS as a multidimensional risk assessment tool capable of predicting not only mortality but also hospital-acquired infections and hypoglycemia, citing its widespread bedside use as a practical advantage for early intervention.^[26]^ Additionally, combining BS with demographic and clinical comorbidity data may enhance its predictive accuracy. Giovannoni et al recommended supplementing BS with factors such as age, incontinence, and catheter use to improve risk stratification for pressure ulcers.^[27]^ Utilizing MIMIC-IV data, our study confirms and expands upon earlier research by showing that reduced BS is independently linked to higher 28-day ICU and 90-day in-hospital mortality in IS patients. Notably, the use of rigorous PSM strengthens the robustness of this association in a focused neurocritical care cohort. Collectively, these findings support the integration of BS into routine ICU assessments as a practical tool for early risk stratification and targeted intervention across diverse critical care populations.

Compared to the similar study by Tang et al, which also identified the BS as a predictor of 30-day mortality in ICU patients with IS, our investigation advances the evidence by employing a more rigorous methodological framework and addressing longer-term prognostic outcomes.^[15]^ While the study of Tang et al involved a larger, highly heterogeneous cohort without employing PSM to account for baseline differences between high- and low-risk groups. As a result, the observed associations may have been confounded by imbalances in age, comorbidities, and disease severity. In contrast, our study, although smaller in sample size, utilized robust PSM to generate well-balanced cohorts, thereby enhancing internal validity. We also accounted for a wider range of covariates, including various severity scores (SOFA, APS-III, SAPS-II, and OASIS), which were either not considered or insufficiently controlled in the prior study. Importantly, while Tang et al focused solely on 30-day mortality, we extended follow-up to assess both 28-day ICU and 90-day in-hospital mortality, offering a more comprehensive evaluation of short- and intermediate-term outcomes. Furthermore, our analysis examined the prognostic value of individual Braden subscores and conducted stratified analyses across key clinical subgroups, providing novel insights into the specific components of BS that may contribute to mortality risk. These methodological improvements enhance the clinical relevance and applicability of our findings, reinforcing the BS as a valuable tool for risk stratification in neurocritical care.

This study has several limitations. First, its retrospective design based on the MIMIC-IV database limits the ability to infer causality. Although multivariate adjustments and subgroup analyses were performed, residual confounding cannot be excluded. In addition, key clinical variables, such as sepsis markers like procalcitonin and positive cultures (blood or urine), IS subtypes and severity (National Institutes of Health Stroke Scale), time of symptom onset, treatment information (thrombolysis, thrombectomy, and decompressive craniectomy), and causes of death, were unavailable, restricting the granularity of risk adjustment. Second, the analysis relied solely on the BS recorded at ICU admission; potential prognostic insights from dynamic changes in BS during hospitalization were not assessed. Third, the accuracy and consistency of BS assessments could not be verified due to possible inter-observer variability in nursing documentation, a common limitation in retrospective analyses. Lastly, despite the relatively large sample size, the study’s reliance on a single database may restrict the generalizability of the findings to wider stroke populations. Prospective multicenter studies with standardized BS assessments and longer follow-up periods are necessary to validate and build upon these findings.

## 5. Conclusion

In conclusion, this study demonstrates that the BS serves as a valuable prognostic indicator of mortality in patients with IS, with potential implications for personalized clinical management. Nonetheless, limitations including its retrospective design, the absence of key clinical and treatment-related variables, and reliance on a single-center dataset warrant cautious interpretation. Prospective, multicenter studies are necessary to validate and generalize these findings.

## Acknowledgments

The authors would like to thank the team of the Laboratory for Computational Physiology from the Massachusetts Institute of Technology for the development and maintenance of the MIMIC‐IV databases.

## Author contributions

**Conceptualization:** Yeliang Mei, Zhenyu Fan.

**Formal analysis:** Yeliang Mei.

**Investigation:** Yeliang Mei, Jiaqi Zi.

**Methodology:** Yeliang Mei.

**Project administration:** Yeliang Mei.

**Resources:** Zhenqiang Liu, Jinyu Huang.

**Supervision:** Zhenyu Fan.

**Visualization:** Jiaqi Zi.

**Writing – original draft:** Yeliang Mei, Jiaqi Zi.

**Writing – review & editing:** Yeliang Mei.

## Supplementary Material
