# Supplementary material for: Association between Braden Score and all-cause mortality in critically ill patients with ischemic stroke: A MIMIC-IV retrospective analysis
Source: Medicine (Baltimore). 2025 Sep 26;104(39):e44674. doi: 10.1097/MD.0000000000044674 (PMC13593027; doi:10.1097/MD.0000000000044674)
Supplement: Supplementary file 1 [file medi-104-e44674-s001.docx]

Supplementary table 1. Braden score scale.

| Item | Level 1 | Level 2 | Level 3 | Level 4 |
| --- | --- | --- | --- | --- |
| Sensory perception | Completely limited: unresponsive (does not moan, flinch or grasp) to painful stimuli because of diminished level of consciousness or sedation OR a limited ability to feel pain over most of body surface | Very limited: responds only to pain, cannot communicate discomfort except by moaning or restlessness, OR has sensory impairment limiting ability to feel pain/discomfort over half of body | Slightly limited: responds to verbal commands but cannot always communicate discomfort or need to be turned, OR has sensory impairment limiting ability to feel pain/discomfort in 1-2 extremities | No impairment: responds to verbal commands, able to feel and express pain/discomfort |
| Moisture | Constantly moist: skin is kept moist almost constantly by perspiration, urine, etc; dampness detected every time patient is moved/turned | Very moist: skin is often but not always moist; linen must be changed at least once a shift | Occasionally moist: requiring extra linen change approximately once a day | Rarely moist: skin usually dry, linen only requires changing at routine intervals |
| Activity | Bedfast: confined to bed | Chairfast: ability to walk severely limited or non-existent; cannot bear own weight and/or must be assisted into chair/wheelchair | Walks occasionally: during day but for very short distances with or without assistance; spends majority of shift in bed/chair | Walks frequently: walks outside room ≥2x/day and inside room at least once every 2 hrs during waking hours |
| Mobility | Completely immobile: does not make even slight changes in body or extremity position without assistance | Very limited: makes occasional slight changes in body or extremity position but unable to make frequent or significant changes independently | Slightly limited: makes frequent though slight changes in body or extremity position independently | No limitation: makes major and frequent changes in position without assistance |
| Nutrition | Very poor: never eats complete meal; rarely eats >1/3 of any food offered; eats ≤2 servings protein (meat or dairy) daily; takes fluids poorly; does not take liquid dietary supplement; or is NPO and/or maintained on clear liquids or IV for >5 days | Probably inadequate: rarely eats a complete meal and generally eats only about one-half of any food offered; protein intake includes only three servings of meat or dairy products per day; occasionally takes a dietary supplement OR receives less than optimum amount of liquid diet or fed by tube | Adequate: eats over half of most meals; eats 4 servings protein (meat or dairy) daily; occasionally refuses meal but will usually take supplement when offered; or is on a tube feeding/TPN regimen which probably meets most of nutritional needs | Excellent: eats most of every meal; never refuses a meal; usually eats ≥4 servings of meat and dairy products; occasionally eats between meals; supplementation not required supplementation |
| Friction/shear | Problem: requires moderate to maximum assistance in moving; complete lifting with- out sliding against sheets is impossible; frequently slides down in bed or chair, requiring frequent repositioning with maximum assistance; spasticity, contractures or agitation leads to almost constant friction | Potential problem: moves feebly or requires minimum assistance; during a move, skin probably slides to some extent against sheets, chair, restraints or other devices; maintains relatively good position in chair or bed most of the time but occasionally slides down | No apparent problem: moves in bed/chair independently, has sufficient muscle strength to lift up completely during move, maintains good position in bed/chair | / |

Note:

The Braden score scale is a standardized tool used by healthcare professionals to assess a patient's risk of developing pressure injuries. It assesses six key areas: Sensory, Moisture, Activity, Mobility, Nutrition, and Friction/Shear. Scores in most categories range from 1 to 4, except for Friction/Shear, which has a maximum score of 3. The total score can range from 6 to 23, with lower scores indicating a higher risk of pressure injuries development. By using this scale, healthcare professionals can systematically identify patients at increased risk and implement appropriate preventive interventions.

Supplementary table 2. Missing number for risk variables and outcome variables.

| Variable | Missing number | Percent | Total |
| --- | --- | --- | --- |
| Gender | 0 | 0 | 2178 |
| Age | 0 | 0 | 2178 |
| LOS Hospital | 0 | 0 | 2178 |
| LOS ICU | 0 | 0 | 2178 |
| 28‐day ICU mortality | 0 | 0 | 2178 |
| 90‐day hospital mortality | 0 | 0 | 2178 |
| Braden | 0 | 0 | 2178 |
| Sensory Perception | 0 | 0 | 2178 |
| Skin Moisture | 0 | 0 | 2178 |
| Activity Level | 0 | 0 | 2178 |
| Mobility | 0 | 0 | 2178 |
| Nutrition | 0 | 0 | 2178 |
| Friction Shear | 0 | 0 | 2178 |
| Heart failure | 0 | 0 | 2178 |
| Myocardial infarct | 0 | 0 | 2178 |
| Arterail fibrillation | 0 | 0 | 2178 |
| Respiratory failure | 0 | 0 | 2178 |
| Diabetes | 0 | 0 | 2178 |
| Paraplegia | 0 | 0 | 2178 |
| Renal disease | 0 | 0 | 2178 |
| Sepsis | 0 | 0 | 2178 |
| Cancer | 0 | 0 | 2178 |
| Stroke | 0 | 0 | 2178 |
| Hemoglobin | 0 | 0 | 2178 |
| Platelet | 3 | 0.14 | 2178 |
| RBC | 0 | 0 | 2178 |
| WBC | 0 | 0 | 2178 |
| Serum creatinine | 37 | 1.7 | 2178 |
| Glucose | 47 | 2.16 | 2178 |
| Sodium | 37 | 1.7 | 2178 |
| SOFA | 0 | 0 | 2178 |
| APS-III | 0 | 0 | 2178 |
| SAPS-II | 0 | 0 | 2178 |
| OASIS | 0 | 0 | 2178 |
| GCS | 0 | 0 | 2178 |
| CCI | 0 | 0 | 2178 |

Abbreviation: APS-III, acute physiology score III; CCI, Charlson comorbidity index; GCS, Glasgow coma scale; ICU, intensive care unit; LOS, Length of Stay; OASIS, oxford acute severity of illness score; RBC, red blood cell; SAPS-II, simplifed acute physiological score II; SOFA, sequential organ failure assessment; WBC, white blood cell.

Supplementary table 3. Characteristics and outcomes of participants categorized by Braden scores after propensity score matching.

| Variable | Total (n=1282) | Braden score low (n=641) | Braden score high (n=641) | p-value |
| --- | --- | --- | --- | --- |
| Personal characteristics |  |  |  |  |
| Age (years) | 70.83(60.10,81.10) | 71.13(60.47,81.85) | 70.49(59.43,80.44) | 0.392 |
| Gender (%) |  |  |  | 0.544 |
| Female | 642(50.08) | 327(51.01) | 315(49.14) |  |
| Male | 640(49.92) | 314(48.99) | 326(50.86) |  |
| Scores |  |  |  |  |
| SOFA | 3.00(2.00,5.00) | 3.00(2.00,5.00) | 3.00(1.00,5.00) | 0.181 |
| APS-III | 36.00(28.00,46.00) | 36.00(28.00,47.00) | 35.00(27.00,46.00) | 0.315 |
| SAPS-II | 31.00(25.00,39.00) | 32.00(26.00,38.00) | 31.00(25.00,39.00) | 0.072 |
| OASIS | 30.00(26.00,35.00) | 30.00(26.00,35.00) | 30.00(26.00,35.00) | 0.348 |
| GCS | 14.00(12.00,15.00) | 14.00(12.00,15.00) | 14.00(13.00,15.00) | 0.163 |
| CCI | 6.00(4.00,8.00) | 6.00(4.00,8.00) | 6.00(4.00,8.00) | 0.869 |
| Comorbidities |  |  |  |  |
| Heart failure (%) |  |  |  | 0.957 |
| No | 970(75.66) | 484(75.51) | 486(75.82) |  |
| Yes | 312(24.34) | 157(24.49) | 155(24.18) |  |
| Myocardial infarct (%) |  |  |  | 0.824 |
| No | 1086(84.71) | 545(85.02) | 541(84.40) |  |
| Yes | 196(15.29) | 96(14.98) | 100(15.60) |  |
| Arterail fibrillation (%) |  |  |  | 0.613 |
| No | 776(60.53) | 383(59.75) | 393(61.31) |  |
| Yes | 506(39.47) | 258(40.25) | 248(38.69) |  |
| Respiratory failure (%) |  |  |  | 0.886 |
| No | 1071(83.54) | 534(83.31) | 537(83.78) |  |
| Yes | 211(16.46) | 107(16.69) | 104(16.22) |  |
| Diabetes (%) |  |  |  | 0.867 |
| No | 892(69.58) | 448(69.89) | 444(69.27) |  |
| Yes | 390(30.42) | 193(30.11) | 197(30.73) |  |
| Paraplegia (%) |  |  |  | 1.000 |
| No | 579(45.16) | 289(45.09) | 290(45.24) |  |
| Yes | 703(54.84) | 352(54.91) | 351(54.76) |  |
| Renal disease (%) |  |  |  | 0.714 |
| No | 1060(82.68) | 533(83.15) | 527(82.22) |  |
| Yes | 222(17.32) | 108(16.85) | 114(17.78) |  |
| Sepsis (%) |  |  |  | 0.623 |
| No | 1174(91.58) | 584(91.11) | 590(92.04) |  |
| Yes | 108( 8.42) | 57( 8.89) | 51( 7.96) |  |
| Laboratory tests |  |  |  |  |
| WBC (10^9^/L) | 10.10(7.90,13.20) | 10.50(8.20,13.60) | 9.60(7.50,12.60) | 0.645 |
| RBC (10^9^/L) | 3.93(3.39,4.36) | 3.88(3.38,4.35) | 3.96(3.40,4.39) | 0.217 |
| Platelet (10^9^/L) | 204.50(157.00,259.00) | 201.00(157.00,256.00) | 207.00(155.00,263.00) | 0.663 |
| Haemoglobin (g/dL) | 11.70(10.00,13.10) | 11.60(10.00,13.10) | 11.80(10.10,13.20) | 0.243 |
| Sodium (mEq/L) | 140.00(137.00,142.00) | 140.00(137.00,142.00) | 139.55(137.00,142.00) | 0.534 |
| Serum creatinine (mg/dL) | 0.90(0.70,1.20) | 0.90(0.70,1.20) | 0.90(0.70,1.20) | 0.915 |
| Glucose (mg/dL) | 120.00(102.00,149.00) | 123.00(104.00,150.00) | 115.00(100.00,145.00) | 0.276 |
| Outcomes |  |  |  |  |
| ICU LOS (days) | 3.26(1.79,6.96) | 4.09(2.03,8.15) | 2.87(1.53,5.75) | <0.001 |
| Hospital LOS (days) | 8.81(5.00,16.89) | 9.91(6.15,18.80) | 7.77(4.07,15.71) | 0.009 |
| 28‐day ICU mortality (%) |  |  |  | <0.001 |
| Alive | 1196(93.29) | 577(90.02) | 619(96.57) |  |
| Death | 86( 6.71) | 64( 9.98) | 22( 3.43) |  |
| 90‐day hospital mortality (%) |  |  |  | <0.001 |
| Alive | 1139(88.85) | 542(84.56) | 597(93.14) |  |
| Death | 143(11.15) | 99(15.44) | 44( 6.86) |  |

Note: Medians and interquartile ranges (25th and 75th percentiles) were calculated for continuous variables and frequencies and percentages for categorical variables. The Wilcoxon rank sum test was used to compare group differences for continuous variables and Chi‐squared tests for categorical variables.

Abbreviation: APS-III, acute physiology score III; CCI, Charlson comorbidity index; GCS, Glasgow coma scale; ICU, intensive care unit; LOS, Length of Stay; OASIS, oxford acute severity of illness score; RBC, red blood cell; SAPS-II, simplifed acute physiological score II; SOFA, sequential organ failure assessment; WBC, white blood cell.
